# Kinetic modeling and exploratory numerical simulation of chloroplastic starch degradation

**DOI:** 10.1186/1752-0509-5-94

**Published:** 2011-06-18

**Authors:** Ambarish Nag, Monte Lunacek, Peter A Graf, Christopher H Chang

**Affiliations:** 1 Computational Sciences Center, National Renewable Energy Laboratory, 1617 Cole Boulevard, MS 1608, Golden, CO 80401, USA

## Abstract

**Background:**

Higher plants and algae are able to fix atmospheric carbon dioxide through photosynthesis and store this fixed carbon in large quantities as starch, which can be hydrolyzed into sugars serving as feedstock for fermentation to biofuels and precursors. Rational engineering of carbon flow in plant cells requires a greater understanding of how starch breakdown fluxes respond to variations in enzyme concentrations, kinetic parameters, and metabolite concentrations. We have therefore developed and simulated a detailed kinetic ordinary differential equation model of the degradation pathways for starch synthesized in plants and green algae, which to our knowledge is the most complete such model reported to date.

**Results:**

Simulation with 9 internal metabolites and 8 external metabolites, the concentrations of the latter fixed at reasonable biochemical values, leads to a single reference solution showing β-amylase activity to be the rate-limiting step in carbon flow from starch degradation. Additionally, the response coefficients for stromal glucose to the glucose transporter k_cat _and K_M _are substantial, whereas those for cytosolic glucose are not, consistent with a kinetic bottleneck due to transport. Response coefficient norms show stromal maltopentaose and cytosolic glucosylated arabinogalactan to be the most and least globally sensitive metabolites, respectively, and β-amylase k_cat _and K_M _for starch to be the kinetic parameters with the largest aggregate effect on metabolite concentrations as a whole. The latter kinetic parameters, together with those for glucose transport, have the greatest effect on stromal glucose, which is a precursor for biofuel synthetic pathways. Exploration of the steady-state solution space with respect to concentrations of 6 external metabolites and 8 dynamic metabolite concentrations show that stromal metabolism is strongly coupled to starch levels, and that transport between compartments serves to lower coupling between metabolic subsystems in different compartments.

**Conclusions:**

We find that in the reference steady state, starch cleavage is the most significant determinant of carbon flux, with turnover of oligosaccharides playing a secondary role. Independence of stationary point with respect to initial dynamic variable values confirms a unique stationary point in the phase space of dynamically varying concentrations of the model network. Stromal maltooligosaccharide metabolism was highly coupled to the available starch concentration. From the most highly converged trajectories, distances between unique fixed points of phase spaces show that cytosolic maltose levels depend on the total concentrations of arabinogalactan and glucose present in the cytosol. In addition, cellular compartmentalization serves to dampen much, but not all, of the effects of one subnetwork on another, such that kinetic modeling of single compartments would likely capture most dynamics that are fast on the timescale of the transport reactions.

## Background

Insolation is the dominating contributor to a sustainable terrestrial energy balance, whether directly captured or transformed into secondary sources such as wind or biomass. Plants have evolved to use this resource to provide themselves with the low-potential carbon necessary for growth by splitting water and fixing carbon dioxide, a known greenhouse gas. During the light photosynthetic reactions, more carbon can be fixed than can be productively marshalled for growth, and cells store this excess carbon in compact polymers such as starch. Chloroplastic starch is stored in the form of granules [1] that consist of both linear and branched polymers of glucose; the process of phase transfer between the granule and the aqueous chloroplast stroma is not known in great detail, although phosphorylation by glucan water dikinase [2,3] and phosphoglucan water dikinase [4,5] may be involved. Amylopectin, the major component of starch, is moderately branched, comprises the majority of starch mass, and is responsible for the crystallinity of starch granules. Essentially unbranched amylose, on the other hand, is amorphous and constitutes up to 30% by weight of starch, depending on culture status [6]. The backbone of both polymers arises from α-1,4 glycosidic bonds; the α-1,6 branches of amylopectin occur every 24 to 30 glucose units.

Much remains unknown about the biochemical pathway for starch degradation in plants and algae. Smith, *et al*. have proposed a pathway of starch degradation in *Arabidopsis thaliana *leaves, whereby starch is released from the granule in a soluble form, then debranched to yield soluble linear glucans in the chloroplast stroma [1,7]. Two mutually alternative degradation pathways can then cleave the linear glucans. In the first, chloroplastic glucan phosphorylase catalyzes the phosphorolytic release of glucose-1-phosphate [8,9], which is cleaved to triose phosphate and the latter antiported in exchange with cytosolic inorganic phosphate [10]. In the second, β-amylase hydrolyzes linear glucans to maltose and maltotriose. Recent results show this second pathway to be more usual in the *Arabidopsis thaliana *chloroplast [1,11,12]. β-amylase releases maltose from the non-reducing ends of linear glucan chains at each catalytic turnover [1], but cannot act on chains of less than four glucosyl units, leading to maltotriose as a by-product of β-amylolytic degradation. Although generally functioning as a predominantly hydrolytic enzyme *in vivo*, β-amylase from sweet potato has been shown to catalyze the condensation of maltose to maltotetraose *in vitro *[13].

Once liberated, maltose and maltotriose can enter chloroplastic and cytosolic carbon pathways. Strong experimental evidence suggests that maltose is exported from the chloroplast stroma to the cytosol by the MEX1 transporter [14]. Cytosolic transglucosidase DPE2 [15-17] can split the transported sugar, glucosylating a soluble endogenous acceptor [1] and freeing glucose. A possible candidate for this acceptor is a soluble arabinogalactan [18,19] that serves as a glucosylation substrate of cytosolic glucan phosphorylase *in vitro *with glucose-1-phosphate as the donor [1,18]. DPE2 and reversible glucan phosphorylase acting together may therefore result in maltose-derived glucose-1-phosphate. The maltotriose product of chloroplastic β-amylase may be acted upon by a disproportionating enzyme (α-1,4 glucanotransferase, DPE1) [20] catalyzing the disproportionation of two maltotriose molecules to glucose and maltopentaose, that can in turn be cleaved by β-amylase to produce maltotriose to re-enter the disproportionation reaction and maltose to be transported out of the stroma. At the catabolic end of starch degradation, cytosolic glucose is phosphorylated at C6 by hexokinase [1,21] for entry into general cellular metabolism.

There currently exists no mathematical model of starch degradation pathways that includes the details discussed in the previous paragraphs. We therefore report the development of a detailed ordinary differential equation (ODE) model that includes most of the biochemical reactions discussed above, and detailed kinetic mechanisms captured from the scientific literature, presumed by direct comparisons, or postulated within the range of characterized mechanisms and parameter values. This approach of hypothesizing unknown values differs from flux balance [22,23] or energy balance [24,25] approaches, where extrema in carefully crafted (*i.e*., the setting of lower and upper bounds, and construction of the objective function(s)) flux spaces are evaluated. Almost all biochemical reactions are catalyzed by enzymes that can saturate, respond non-linearly to changes in metabolite concentrations, and comprise components of a reaction network capable of dynamic evolution outside of the steady-state assumption. Although insightful results have been obtained from several studies [26-28] on specific metabolic pathways incorporating known enzyme kinetics, the most promising features of the current modeling approach are a greater understanding of *potential *nonlinear network dynamics, and the possibility of characterizing the high-dimensional space of metabolic responses with respect to enzyme concentrations and parameters using modern high-performance computing.

**Figure 1:**
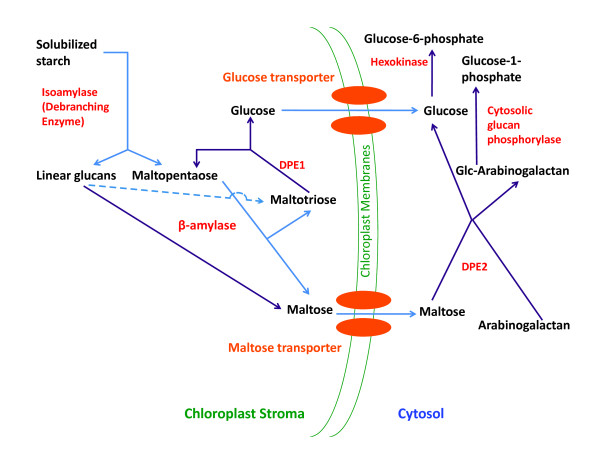
**Biochemical and transport reactions in current starch degradation model**. The carbon flux in the starch degradation pathway from solubilised starch in the chloroplast stroma to hexose phosphates in the cytosol has been schematically represented in this figure. The pathway consists of seven enzyme catalyzed reactions and two transport reactions. The different types of species in this figure have been color-coded. The metabolite species are shown in black; enzymes are in red and transporters in orange; and, irreversible and reversible reactions are shown as blue and purple arrows, respectively. The dashed arrow showing maltotriose formation from linear glucans reflects that this reaction is substoichiometric, occurring only once per odd-length chain.

The current model focuses on steady-state solubilized starch catabolism. A starch degradation model previously postulated [1] did not include the effective competition for β-amylase of maltose condensation to maltotetraose [13]; the effects of this alternative sink reaction on β-amylase turnover have been included in the present model by inclusion of an inhibition term as previously formulated by Shiraishi and coworkers [29]. The biochemical reactions in the current starch degradation model have been schematically represented in Figure 1. Enzyme kinetic parameters are taken from reported values, calculated from Haldane relations [30], or assigned reasonable values within the relatively limited range of known values for the particular parameter in question.

## Results

### Model content

The model detailed above contains 17 metabolites, 6 enzymes, 2 transporter proteins, and 3 inhibitors that participate in 9 reactions characterized by 63 enzyme kinetic and binding parameters. Eight metabolites are at the boundary of the system and therefore act as systemic parameters and are referred to as "external metabolites"; the remaining nine are free, and called "internal metabolites". The model encompasses the chloroplast stroma, cytosol, and chloroplast intermembrane space containing two transporter proteins linking the stromal and chloroplastic metabolite pools. The intermembrane space impacts simulations only by defining the volume affecting these transporters' concentrations.

The initial concentration of the intermediate linear linkage group Starch_db__CS was set to zero in all calculations. The pH value of the cytosol is assumed to be 7, so that the proton concentration in the cytosol is fixed at 0.1 μM. All other internal and external metabolites take on concentrations in the molar to nanomolar range. Although extremes of this range are of questionable physiological significance, two conciliatory considerations apply. First, the dynamical system is dictated primarily by the controlling equations, and secondarily by the particular point in concentration space that the system occupies. Thus, the system will evolve toward a steady state in as robust a manner as the underlying phase space permits, dissipating or accumulating excess mass from the external metabolite baths as necessary. Second, we are explicitly interested in the fixed point(s) of the dynamical space arising from the reaction network topology and the structure of the kinetic equations, such that concentrations beyond biologically relevant bounds are desirable to characterize the possible behaviors of the system.

Six external metabolites were held at fixed concentrations to reflect their coupling to a homeostatic cellular reservoir: chloroplastic starch glucosyl residues, and the cytosolic pools of ATP, ADP, phosphate, glucose-1-phosphate, and glucose-6-phosphate. Starch and polymerized glucosyl units were also modeled as external metabolites to reflect a large starch reservoir, such as would be relevant to the transition between photosynthetic starch accumulation and biofuel-producing fermentative metabolism. Three species (cytosolic reduced glutathione, cytosolic glucose-1,6-bisphosphate, and cytosolic 2,3-bisphosphoglycerate) act as hexokinase inhibitors but do not otherwise participate in any reaction; they are therefore classified as parameters. It should be noted that from a mathematical and dynamical perspective, external metabolites are also model parameters. Our chosen distinction between "external metabolites" and parameters (the three metabolites above as well as kinetic and binding constants) is based on participation or non-participation as a reactant or product in a modeled reaction, and so is less operational than chemically ontological. Enzyme and transporter protein concentrations are held constant to reflect a particular metabolic state.

### States explored

Three primary models are explored in detail. The "reference" model or state is described in detail in the Methods section, and is comprised of a single best estimate of concentrations, kinetic parameter values, and protein concentrations. Two other models derived from this reference system were also analyzed to explore the robustness of the reference system to perturbations of kinetic parameters or enzyme concentrations. The first decreased  and  10-fold, and is named the "parameter-perturbed" model or state. The second increased the reference concentrations of β-amylase and MEX two-and 10-fold, respectively, and is named the "enzyme-perturbed" model or state. In addition to these three models, a space of models differing in either initial concentrations of internal metabolites or fixed concentrations of external metabolites was generated by 2-way sampling of 14 concentrations (8 internal and 6 external), thus yielding a body of 2^14 ^= 16,384 individual simulations that is analyzed and discussed separately.

### Response coefficients with respect to kinetic and binding parameters

**Figure 2:**
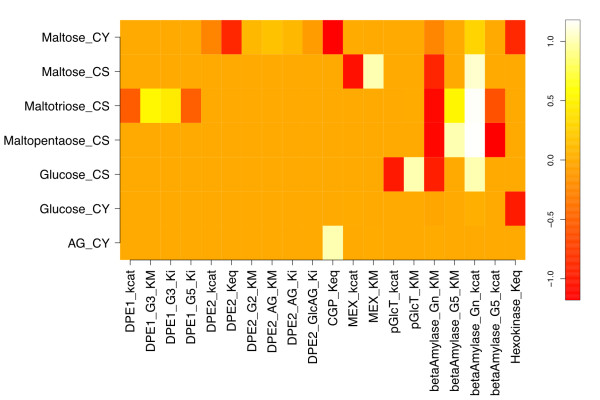
**Metabolite response coefficients with respect to kinetic and binding parameters for the reference model**. The matrix of response coefficients with respect to the enzyme kinetic and binding parameters in the starch degradation model at the reference steady state is shown as a heat map representation. Only coefficients with at least one magnitude ≥ 0.05 are included.

Response coefficients quantify the sensitivity of steady-state variables to variation in model parameters, and so pertain to a particular steady state [31]. Here we focus on the response of steady-state metabolite concentrations only, and include as model parameters both kinetic constants and enzyme concentrations. For the reference steady state, the concentration response coefficients with respect to variations in kinetic and binding parameters in the model are presented as a heat map representation in Figure 2. The elements *x_im _*of the matrix in Figure 2 are the response coefficients  for each internal metabolite species *i *at steady state, with respect to each parameter *m*, excepting intermediate species Starch_db__CS (*S_db_*) for reasons discussed below.

The β-amylase k_cat _() has the most response coefficients above zero, with the starch Michaelis constant for the same reaction () showing a similar pattern below zero. This inverse relationship is expected due to the definitions of these kinetic parameters. This responsiveness extends through the plastidic starch degradation products maltose and maltotriose, weakens at cytosolic maltose, and is essentially zero for cytosolic glucose and arabinogalactan.

**Figure 3:**
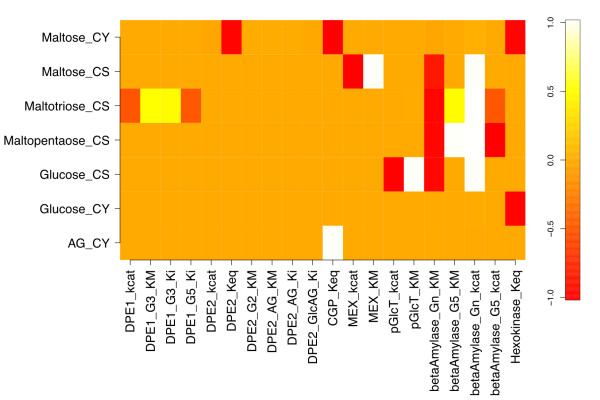
**Metabolite response coefficients with respect to kinetic and binding parameters for the parameter-perturbed model**. The response coefficients in Figure 4 are recalculated from the simulation results obtained on reducing both the β-amylase turnover number for starch hydrolysis () and the maltose exporter (MEX) Michaelis constant for maltose transport () by a factor of 10 compared to the corresponding values for Figure 2. The change in these two kinetic parameters yields a new steady state and therefore a different set of values for the response coefficients.

**Figure 4:**
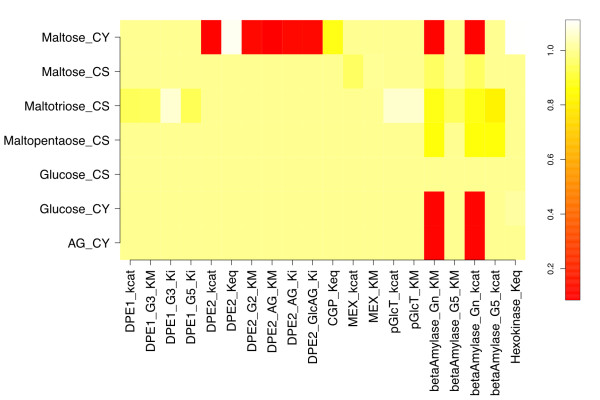
**Ratios of parameter-perturbed and reference response coefficients**. The ratios of each response coefficient in Figure 3 to the corresponding coefficient in Figure 2 are shown. The ratio is set to unity if both response coefficients are less than 10^-3^.

To evaluate the sensitivity of the model's response to the particular kinetic parameters used, a perturbed model with the β-amylase starch  and MEX  decreased by 10-fold ("parameter-perturbed" model) was simulated to steady state, and yielded the response coefficients shown in Figure 3. These parameters were chosen based on β-amylase's role in carbon flux limitation, and MEX's role in coupling the chloroplastic and cytosolic compartments. None of the significant response coefficients in Figure 3 differ in sign from their counterparts in Figure 2. The ratios of parameter-perturbed to reference coefficients are mapped in Figure 4. Response coefficients less than a cutoff value (10^-3^) were considered as zero, and the ratio of two such coefficients replaced by 1.0 to avoid numerical noise from dividing small numbers. Figure 4 shows that the response coefficients of cytosolic maltose with respect to β-amylase  and , and five DPE2 kinetic parameters (*k_cat_*, *K_M _*for maltose and arabinogalactan, and *K_i _*for arabinogalactan and glucosylated AG) are reduced in magnitude relative to those for the reference steady state. However, maltose responsiveness to the DPE2 and hexokinase equilibrium constants increases, due to more negative values upon perturbation. Cytosolic glucose and arabinogalactan also show decreased response with respect to  and . Chloroplastic maltopentaose response coefficients exhibit smaller reductions versus ,  and . Thus, most changes to concentration responsiveness are decreased upon the coupled decrease in β-amylase turnover and increase in maltose export from the stroma, primarily relative to β-amylase starch degradation kinetics and cytosolic maltose cleavage, with DPE2 and hexokinase *K_eq _*being exceptions. This pattern primarily reflects the decreased starch cleavage rate, leading to roughly 100-fold lower stromal maltose (240 vs. 2.3 μM), but approximately the same cytosolic maltose (110 vs. 130 mM) concentrations, lower flux through the DPE2 reaction, and a greater sensitivity to the direction of the DPE2 and hexokinase reactions. No ratio is greater than 1.1 or less than 0.08.

### Row (ρ) and column norms (κ) of the parametric response coefficient matrix

**Figure 5:**
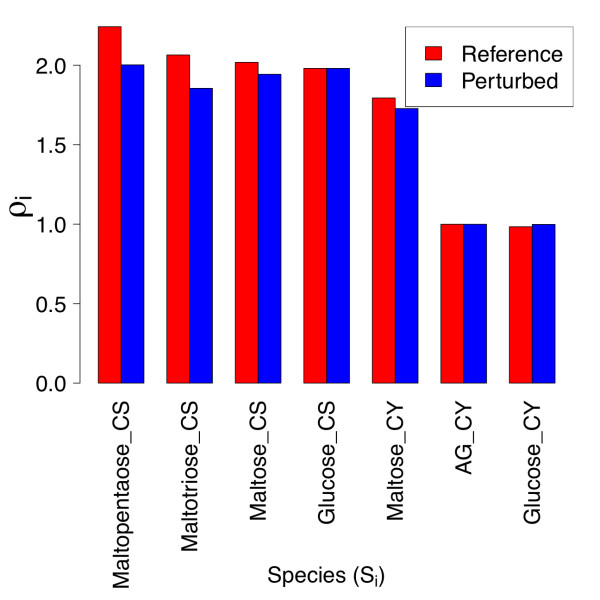
**Metabolite sensitivities of individual metabolites versus kinetic and binding parameters for reference and parameter-perturbed states**. The collective sensitivity *ρ_i _*of individual metabolites to all kinetic and binding parameters are shown for the reference state, and a model with 10-fold reduced β-amylase  and MEX  values. The quantities *ρ_i _*are defined as the row norms of individual response coefficients, as discussed in the text.

**Figure 6:**
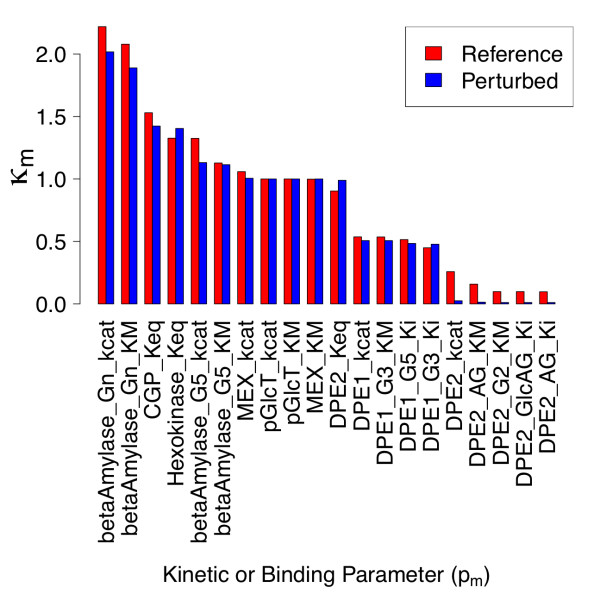
**Collective sensitivities with respect to individual kinetic and binding parameters for reference and parameter-perturbed states**. Collective sensitivity *κ_m _*of all steady state metabolite concentrations with respect to individual kinetic or binding parameters are shown for the reference state, and a model with 10-fold reduced values of β-amylase  and MEX . The quantities *κ_m _*are defined as the column norms of individual response coefficients, as discussed in the text.

To evaluate the global sensitivity of specific metabolite concentrations *S_i _*with respect to kinetic and binding parameters *p_m _*in Figure 2, the Euclidean norm of each metabolite's response coefficient vectors were calculated as the root-mean-square of response coefficients along row *i*,

The converse quantity, namely the overall sensitivity of steady-state metabolite concentrations to variation in individual model parameter values *p_m_*, was calculated as the Euclidean norm along column *m*,

For the set of species considered in Figure 2, we show the bar plot of the corresponding *ρ_i _*in Figure 5. The calculated *κ_m _*for each of the kinetic and binding parameters in Figure 2 are shown in Figure 6, which includes only the twenty largest *κ *values. The steady-state concentration of stromal maltopentaose is the most generally sensitive metabolite in the present model, while cytosolic glucosylated arabinogalactan shows the least global sensitivity (not shown). Figure 6 indicates that  and  have the largest aggregate effects on metabolite concentrations at this reference steady state, consistent with these parameters corresponding to the most positive and negative response coefficients in Figure 2.

Despite maltotriose and maltose being sensitive to more kinetic and binding parameters than maltopentaose, maltopentaose has the greatest ρ due to the large magnitudes of the maltopentaose parametric response coefficients for reactions involving the conversion of starch and maltopentaose to maltose and maltotriose. A potential reason for this strong coupling is a positive feedback loop: degradation of starch and maltopentaose by β-amylase yields maltose and maltotriose, two molecules of which disproportionate to yield maltopentaose. The least sensitive metabolite, glucosylated arabinogalactan, is simply a non-transient intermediate for transfer of a glucosyl unit from maltose to phosphate via a soluble arabinogalactan that is regenerated at the end of the transfer. Modification of flux into this reaction can increase or decrease the AG/GlcAG ratio, but the concentration response of either is bounded by the total fixed arabinogalactan concentration available. The response coefficient of greatest magnitude for glucosylated arabinogalactan corresponds to the equilibrium constant for glucosyl transfer, emphasizing the locality of this metabolite's response.

Figure 5 also displays the row norms for the parameter-perturbed steady state. Chloroplastic maltopentaose *ρ* is reduced relative to that for the reference steady state, due to the lower values of the response coefficients of maltopentaose with respect to three β-amylase enzyme kinetic parameters (Figure 4). In spite of the significant reduction in the response coefficients of cytosolic glucose and arabinogalactan versus a number of kinetic and binding parameters for the parameter-perturbed state, the *ρ* values of these two metabolites are comparable between states. This appears to be so because the response coefficients of cytosolic glucose and arabinogalactan are very small in both cases, so large relative changes have little effect on the norm. The *ρ* value for cytosolic maltose is also not significantly altered upon the perturbation considered, due to compensatory changes in magnitudes of *R^maltose^*.

Upon perturbation, the response coefficient column norms with respect to β-amylase *k_cat _*and *K_M _*and several DPE2 kinetic parameters are reduced in magnitude, whereas the column norms with respect to hexokinase and DPE2 equilibrium constants become larger (Figure 6). These trends follow directly from the observations in Figure 4 which have been discussed in detail above.

### Comparison of response coefficients for chloroplastic glucose with respect to kinetic and binding parameters

Because glucose is a precursor for fermentative pathways yielding important potential biofuels, including ethanol and hydrogen, its associated response coefficients are of special interest. The glucose steady-state concentration in the stroma is effectively sensitive to only four kinetic parameters, two having direct proportionality (*i.e*., parameter increase yields metabolite increase), and two inverse proportionality, with all four magnitudes close to 1.  is positive and  is negative, consistent with expectations regarding *k_cat _*and *K_M_*. A similar but inverse relationship is evident in the *k_cat _*and *K_M _*parameters of the glucose transporter, where turnover implies movement of glucose out of the plastid; so, decelerating this transporter's action will increase steady-state chloroplastic glucose concentration. DPE1, maltose transport, and debranching kinetics had little effect on stromal glucose levels.

Rearrangement and integration of the definition for *ρ_i _*above allows us to discover the parametric dependence on *S_i _*as

**Figure 7:**
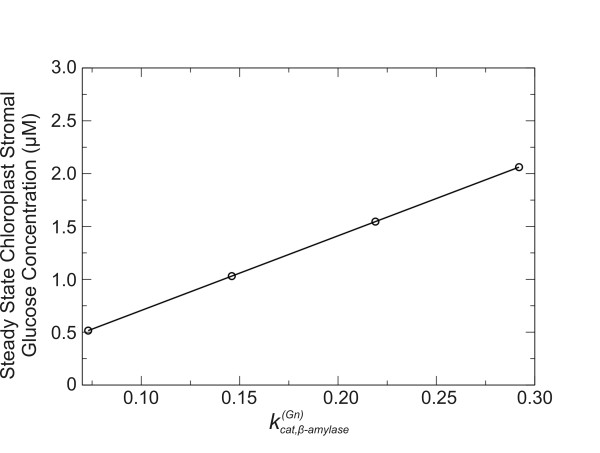
**Variation in simulated steady state concentration of chloroplast stromal glucose with the β-amylase turnover number for starch hydrolysis**. The dependence of the simulated chloroplastic glucose steady state concentration on β-amylase turnover number for starch hydrolysis () is seen to be linear over a range from ~0.03 to 0.3 s^-1^.

In other words, *S_i _*varies as the *ρ*_i_^th ^power of *p_m_*. Because the response coefficient of chloroplastic glucose with respect to  is positive and very close to unity, if  is varied while keeping all other parameters fixed the steady-state concentration of chloroplastic glucose should increase linearly with the parameter value. This prediction is validated by the plot in Figure 7, where the steady-state concentration of glucose in the chloroplast stroma is shown to vary linearly with β-amylase .

### Response coefficients with respect to enzyme and transporter concentrations

**Figure 8:**
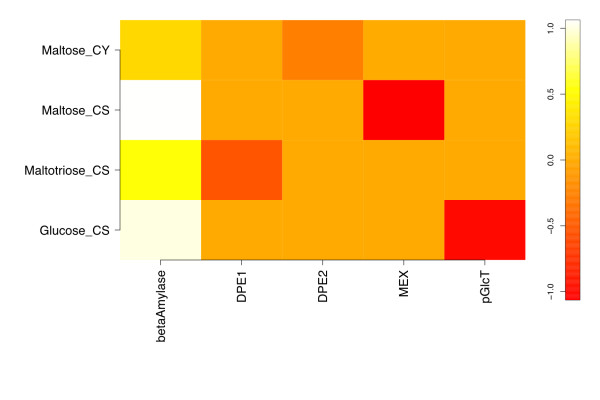
**Metabolite response coefficients with respect to enzyme and transporter levels for the reference model**. Metabolites are along the vertical axis, and enzyme and transporter concentrations along the horizontal axis. Only coefficients with at least one instance of magnitude ≥ 0.05 are shown.

The response coefficients of steady-state metabolite concentrations with respect to enzyme and transporter concentrations for the reference steady state in this study are shown as a heat map in Figure 8. Chloroplastic maltopentaose and cytosolic arabinogalactan, glucosylated arabinogalactan, and glucose show responses near zero, implying lack of sensitivity to any enzyme concentration. Because [*E*] enters linearly into the kinetic equations used, this situation can arise only if (a) enzymes acting on these species are saturated but present in high concentration (*i.e*., have low turnover numbers), such that small changes in enzyme concentration have a proportionally small effect, or (b) turnover is fast enough to keep reactions near equilibrium, so slowing down interconversion slightly by lowering the enzyme concentration does not result in an observable change. Maltopentaose participates in two reactions in the model, both of which are upstream of most other reactions. The response coefficients of maltopentaose with respect to enzymes catalyzing these other reactions are small, as expected for net flux "downstream" and rate limitation "upstream". If downstream enzymes were saturated, a sensitivity of upstream metabolite concentrations to these enzymatic levels would arise. This relationship can be seen in the DPE1-catalyzed disproportionation that yields maltopentaose from maltotriose, and has a higher turnover number (50 s^-1^) than the downstream degradation of maltopentaose by β-amylase (~0.1 s^-1^). As expected, maltopentaose levels have a stronger dependence on the kinetics of the rate-limiting degradation step, and so the maltopentaose steady-state concentration is more sensitive to β-amylase variation than to that of DPE1.

Both arabinogalactan and glucosylated arabinogalactan participate in two reactions that are characterized by high enzymatic turnover in both the forward and reverse directions. Hence, small quantities of the enzymes (DPE2 and cytosolic glucan phosphorylase) catalyzing those two reactions are sufficient to maintain the reactions near equilibrium, resulting in the negligible sensitivities of arabinogalactan and glucosylated arabinogalactan steady-state concentrations to these enzyme concentrations. A similar situation may be assigned to cytosolic glucose, which arrives from the chloroplast stroma via a plastidic glucose transporter with a high transport turnover number. Glucose is also formed directly in the cytosol from maltose and arabinogalactan by DPE2, with high turnover numbers in both the forward and reverse directions. Finally, cytosolic glucose is phosphorylated to glucose-6-phosphate by hexokinase, which has a large forward turnover number. In all three cases, the enzymes and transporters involved are sub-saturated, so that reaction kinetics are relatively insensitive to small changes in enzyme levels.

At the other extreme of sensitivity, the chloroplastic β-amylase exhibits the highest number of large positive response values, with the product maltose having the largest positive sensitivity. This suggests that hydrolysis of linear starch fragments to maltose is limiting the starch degradation flux near the combination of enzyme concentrations and parameters associated with the reference steady state. The β-amylase-associated response coefficients for cytosolic glucose, arabinogalactan, and glucosylated arabinogalactan have their second largest values for any enzyme, despite these metabolites being the most distant variable metabolites from chloroplastic maltose in the reaction network. To the extent that these numerical results represent cellular behavior, β-amylase is a natural target for increasing *in vivo *activity.

**Figure 9:**
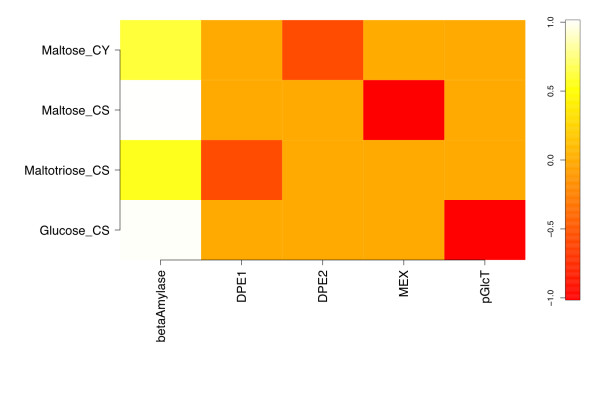
**Metabolite response coefficients with respect to increased enzyme and transporter levels for the enzyme-perturbed model**.

To examine how representative the reference model and steady state are relative to enzyme levels, in Figure 9 are shown the response coefficients in Figure 8 for a model obtained by increasing the β-amylase concentration two-fold and the maltose exporter concentration ten-fold relative to the reference model (the "enzyme-perturbed" model). The perturbation significantly affects only the response coefficients of cytosolic maltose with respect to β-amylase and DPE2 concentrations, changing them 2.14-fold and 2.17-fold, respectively. Higher fluxes of maltose production and transport into the cytosol imply increased dependence of the model system on any reaction that consumes cytosolic maltose to achieve a steady state maltose concentration, so increased DPE2 responsiveness is expected. Given the predictability of the qualitative response and the relatively small change in response coefficients, we consider the reference steady state to be a reasonable representation of the metabolism being explored.

### Row norms of response coefficient matrix with respect to enzyme and transporter concentrations and response coefficients for chloroplastic glucose

**Figure 10:**
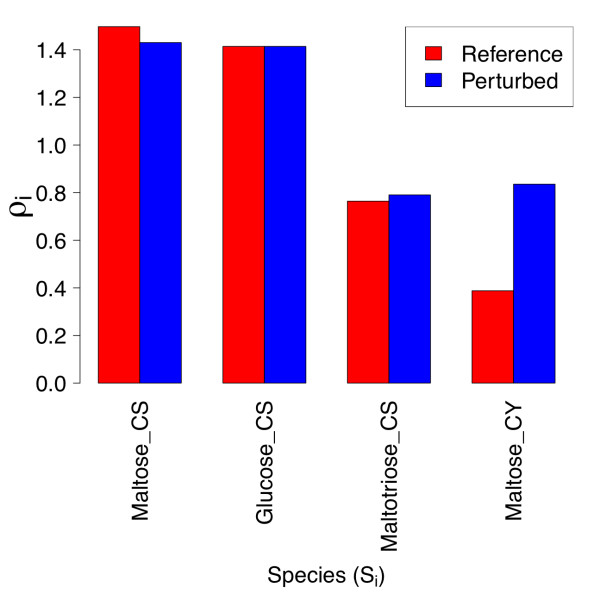
**Collective responses of individual metabolites with respect to all catalyst levels for reference and enzyme-perturbed states**. The collective sensitivity *ρ_i_*, defined as the row norms of individual response coefficients, of individual metabolites *i *to all enzyme and transporter concentrations for the reference and enzyme-perturbed states are shown.

**Figure 11:**
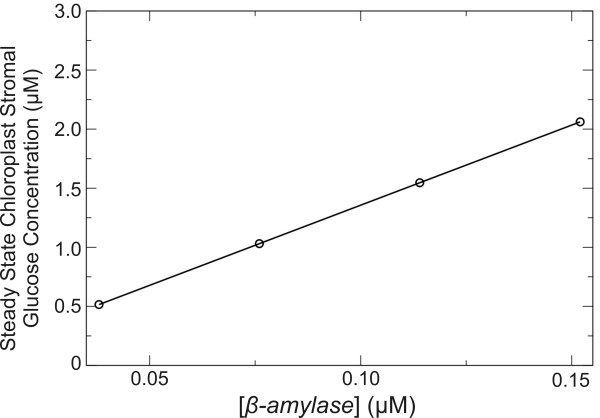
**Dependence of simulated steady state chloroplastic glucose concentration on β-amylase concentration**. The simulated steady state chloroplastic glucose concentration increases linearly with β-amylase concentration in the range 0.038 to 0.152 μM.

The calculated ρ_i _values for the metabolite species in Figure 8 indicate that enzyme and transporter concentrations generally have the greatest impact on the reference steady-state concentrations of chloroplastic maltose and glucose (Figure 10). The two response coefficients of largest magnitude for chloroplastic glucose with respect to β-amylase and the plastidic glucose transporter both had values of 1.0, with the next largest coefficients effectively zero (~10^-18^). To increase the standing concentration of chloroplastic glucose, one would need either to overexpress the chloroplastic β-amylase enzyme, or suppress glucose transport. Simulations with four different concentrations of the β-amylase enzyme show a linear variation on steady-state stromal glucose concentration (Figure 11), consistent with a chloroplastic glucose response coefficient with respect to β-amylase close to unity. For context, experiments suggest that the level of β-amylase activity in leaves of the *hba1 Arabidopsis thaliana *mutant is about six times the corresponding activity level in wild type *Arabidopsis *leaves [32], and that the combined effects of prolonged exposure to light and treatment with sucrose solution can enhance the β-amylase activity in wild-type *Arabidopsis *leaves by approximately a factor of four [33].

For the enzyme-perturbed model, row norms are plotted alongside the reference state values in Figure 10. The only ρ value significantly different from that of the reference steady state is the value for cytosolic maltose, arising primarily from the significant increase (2.17) of  as explained earlier. The contribution to ρ from the 2.14-fold change in  between the two states is minor because this response coefficient is of small magnitude.

### Comparison of κ values from response coefficient matrix with respect to enzyme and transporter concentrations

**Figure 12:**
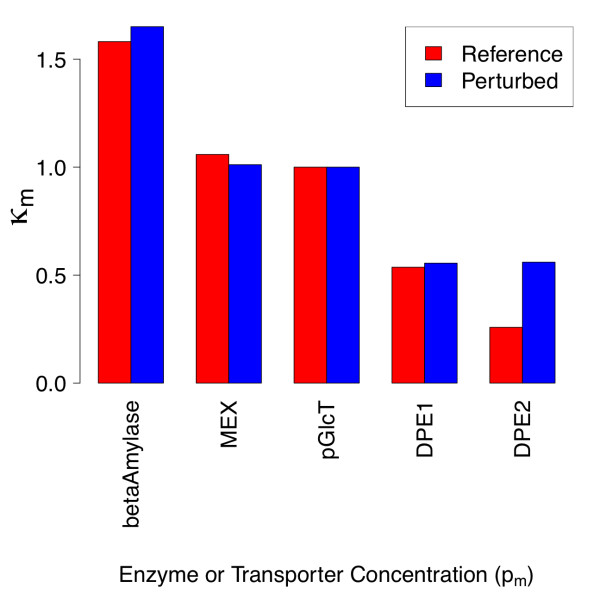
**Collective responses of metabolome with respect to individual catalyst levels for reference state**. The collective sensitivity *κ_m _*of all steady state metabolite concentrations with respect to individual enzyme and transporter concentrations are shown for the reference and enzyme-perturbed states. The quantities *κ_m _*are defined as the column norms of individual response coefficients, as discussed in the text.

The calculated κ values for each of the enzymes or transporters in Figure 8 are shown in Figure 12 for the reference and enzyme-perturbed states. For the former, variation of chloroplastic β-amylase concentration has the maximum aggregate effect on the steady-state concentrations of the small metabolome considered in Figure 8. The plastidic maltose exporter has the second largest effect on the steady-state concentrations of the metabolites, with the major contribution originating from the large negative . For the enzyme-perturbed steady state, the κ_DPE2 _value is the only column norm that differs significantly upon this perturbation. In contrast to the increase in  seen, the analogous increase in the κ_β-amylase _value is insignificant, because the response coefficient of maltose vs. β-amylase is quite small in magnitude such that even a significant relative change has little impact on κ.

### Enumeration of initial metabolic states, convergence thresholds, and clustering analyses

To determine if the starch degradation model constructed was capable of hosting multiple steady states, and to examine the variability of the model behavior with respect to variation of metabolic pool sizes external to the dynamic core, multiple simulations were run from different initial internal, and fixed external, metabolite concentrations (Tables 1 and 2). The concentration of starch is not an independent variable and is related to the concentration of starch glucosyl units. Out of the 17 metabolites, we therefore sampled the initial concentrations of 8 internal metabolites and the fixed concentrations of 6 external metabolites. Sampling *n *initial or fixed concentrations of each of these 14 species yields *n*^14 ^combinations of initial conditions; thus, even for our modest sampling number of *n *= 2, corresponding to initial or fixed concentrations of 0.1 and 1000 μM, 16,384 temporal integrations were needed. For each of 2^6 ^= 64 system parameter combinations, there are thus 2^8 ^= 256 different initial value vectors to test for multi-stationarity. For reasons of parallel load balancing, we elected a fixed integration time of 10^7 ^virtual seconds as a stopping criterion, rather than test for convergence directly. The final metabolite and time derivative data was then post-processed. To analyze the results, we chose to take a global view of all the points generated, as well as to focus on the most highly converged points.

**Table 1:** Concentrations of external metabolites, enzymes, transporters and inhibitors

Species	Symbol	Sub-cellular Location	Concentration (μM)
***External Metabolites***			

H^+^	H+_CY	Cytosol	0.1

Starch(Gn)	Starch_CS	Chloroplast Stroma	0.6*

Starch glucosyl unit	GlcStarch_CS	Chloroplast Stroma	1000

ATP pool	ATPtot_CY	Cytosol	10000

ADP pool	ADPtot_CY	Cytosol	10000

Phosphate pool	Pitot_CY	Cytosol	10000

Glucose-1-phosphate pool	Glc1Ptot_CY	Cytosol	10000

Glucose-6-phosphate pool	Glc6Ptot_CY	Cytosol	10000

***Enzymes***			

β-amylase	β-amylase_CS	Chloroplast Stroma	3.8 × 10^-2^

Isoamylase (debranching enzyme)	ec_3_2_1_68_CS	Chloroplast Stroma	1.475 × 10^-1^

DPE1 enzyme	ec_ 2_4_1_25_CS	Chloroplast Stroma	2

DPE2 enzyme	ec_ 2_4_1_25_CY	Cytosol	2

Cytosolic glucan phosphorylase (CGP)	ec_2_4_1_1_CY	Cytosol	2

Hexokinase	ec_2_7_1_1_CY	Cytosol	10

***Transporters***			

Maltose (MEX)	tc_2_A_84_1_2_CIMS	Chloroplast Intermembrane Space	2

Plastidic Glucose (pGlcT)	tc_2_A_1_1_17_CIMS	Chloroplast Intermembrane Space	20

***Inhibitors***			

Reduced Glutathione	GSH_CY	Cytosol	1000

Glucose-1,6-bisphosphate pool	Glc16BPtot_CY	Cytosol	10000

2,3-bis-phosphoglycerate pool	23BPGtot_CY	Cytosol	10000


The pH of the cytosol is assumed to remain at 7, so that the cytosolic proton concentration is always kept at 0.1 μM. The three species-reduced glutathione, glucose-1,6-bisphosphate and 2,3-bisphosphoglycerate act only as hexokinase inhibitors and are treated as parameters. We have used the mass concentration of the β-amylase (7.83 × 10^-3 ^gm L^-1^) and isoamylase (1.18 × 10^-2 ^gm L^-1^) enzymes mentioned in the caption of Figure 1 in Ref. [29]. For potato β-amylase, the molecular weight is 206 kD, so that we are effectively using a β-amylase concentration of 3.8 × 10^-2 ^μM. Potato isoamylase peptides have a molecular weight of about 80 kDa, so that the effective isoamylase concentration that we are using is effectively 1.475 × 10^-1 ^μM.

*The molecular weight of starch in our model is assumed to be equal to that of Starch B (2.7 × 10^5^) in Ref. [1]. Assuming that starch has a molecular formula of the form H(C_6_H_10_O_5_)_n_(OH), we calculate a value of 1667 for n, which interprets as one molecule starch contains 1667 glucosyl units. Thus 1000 μM of starch glucosyl units is equivalent to 0.6 μM of starch. Hence the concentration of starch is determined by the concentration of starch glucosyl units.

**Table 2:** Initial concentrations of internal metabolites

Internal Metabolites	Symbol	Sub-cellular Location	Initial Concentration (μM)
Debranched starch	Starch_db__CS	Chloroplast Stroma	0

Maltose (G2)	Maltose_CY	Cytosol	10

Maltose	Maltose_CS	Chloroplast Stroma	10

Maltotriose (G3)	Maltotriose_CS	Chloroplast Stroma	100

Maltopentaose (G5)	Maltopentaose_CS	Chloroplast Stroma	1000

Glucose	Glucose_CS	Chloroplast Stroma	10

Glucose	Glucose_CY	Cytosol	10

Arabinogalactan (AG)	AG_CY	Cytosol	10000

Glucosylated Arabinogalactan	GlcAG_CY	Cytosol	10000


Evolution from these initial values gives rise to the reference steady state shown in Figs. 2 and 3. One of the underlying assumptions in our model is that the initial concentration of debranched starch is always zero.

We have therefore clustered the temporal integration end point data at different convergence thresholds, with the following intent. Because each end point represents a trajectory potentially at a different "stage" of evolution, and starting from a different initial point within the metabolite concentration space, we anticipate that selection based on increasingly tight convergences will progressively select for classes of initial points in the metabolite space that lie closer to fixed points of the different systems created by sampling fixed values of the external metabolites. Nevertheless, the long fixed virtual time of the simulations should permit some structure to be observed in the total dataset, and most importantly to classify different clusters based on their associated initial metabolite vectors. If bi-valued samples of the six external metabolites completely determine the phase spaces and each such space has one steady-state fixed point, then one expects the end-point data to fall into 2^6 ^= 64 different clusters, each associated with a different sample of the external metabolite concentrations. Deviations from this null result and multivariate correlations are potentially interesting features of the formulated model system. Centroids with concentration coordinates intermediate between two fixed sample values simply highlight membership within a cluster of trajectories starting from different values of external metabolites, suggesting that despite these different values, the trajectory end points are still near each other in metabolite coordinate space. Correlations among centroid coordinates can elucidate deterministic relationships between metabolites—for example, complete correlation between variables implies that some are dependent on others and hold little explanatory power. Weaker correlations should reflect control relationships among metabolites—an anti-correlation between freely variable × and Y implies an increase of [X] puts negative pressure on [Y].

**Figure 13:**
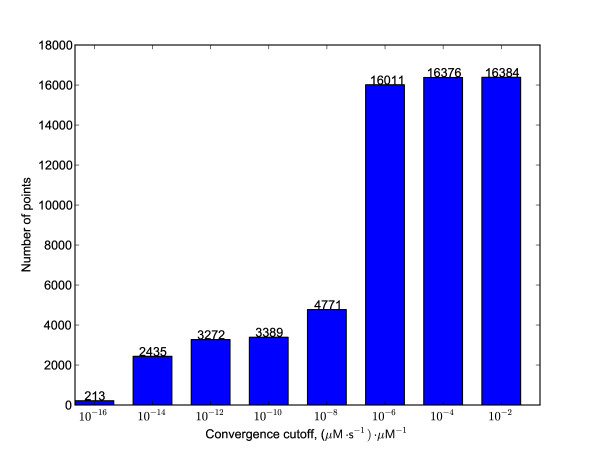
**Cumulative distribution of trajectory number as a function of convergence threshold**. Each integration starting from a unique point in the metabolite space was stopped at 10^7 ^simulated seconds, and the convergence evaluated as described in the text.

In Figure 13 is the cumulative distribution of trajectories at different convergence thresholds. All 16,384 trajectories were found to have converged to less than 10^-2 ^(μM s^-1^) μM. Notable drops in trajectory number can be seen between 10^-6 ^and 10^-8 ^(μM s^-1^) μM, and between 10^-14 ^(μM s^-1^) μM and the tightest cutoff explored here, 10^-16 ^(μM s^-1^) μM. The classes of trajectories found at the more tightly converged ends of these two breakpoints might be expected to differ qualitatively from more loosely converged trajectories and reflect important features of the system. To seek these features, three classes of end points were mapped—all points, those trajectories evolving at less than 10^-8 ^(μM s^-1^) μM^-1^, and those at less than 10^-16 ^(μM s^-1^) μM^-1^. In each case, correlation coefficients between metabolites over the cluster centroids were calculated, in order to highlight co-variation among the species in the model. Data are provided as Additional File 1. Examination of all 16,384 end points shows that stromal maltose, maltotriose, and maltopentaose levels are dictated by the size of the fixed starch pool, with cytosolic maltose levels correlating predominantly with this pool as well. The latter correlates negatively but weakly with the arabinogalactan pool, ATP, and phosphate, and positively with glucosylated arabinogalactan and glucose-1-phosphate. Cytosolic glucose correlation coefficients, though less than 1.0, show a similar correlation pattern similar those of stromal glucose. The magnitude of 0.3 suggests that most of the cytosolic glucose coupling is to species other than starch and maltose oligomers in the stroma; the uniform magnitude of correlation coefficients to the latter suggest that transport between the stroma and the cytosol may decrease the metabolic coupling between the starch repository and cytosolic glucose levels. Arabinogalactan is anti-correlated with cytosolic maltose, GlcAG, and glucose-1-phosphate, and correlated positively with cytosolic glucose and free phosphate. GlcAG correlates negatively with cytosolic glucose, AG, and free phosphate, but positively and weakly with cytosolic maltose, ADP, and glucose-1-phosphate. This pattern can be reasoned from the pathway diagram in Figure 1, with the possible exception of the negative correlation between AG and GlcAG. This latter relationship likely arises due to the conserved pool size of total arabinogalactan—if both varied independently, more GlcAG would push the reversible DPE2 reaction toward AG; since the total pool size is constant, however, more GlcAG must mean less AG. Negative correlations between ATP and cytosolic glucose and maltose are consistent with increases of the latter putting pressure on ATP supply in the hexokinase reaction. Phosphate shows mainly positive correlation with AG and glucose-1-phosphate, and negative with GlcAG, arising from the glucan phosphorylase and DPE2 reactions together with the fixed pool of AG + GlcAG. Glucose-1-phosphate couplings reflect these same relationships. The glucose-6-phosphate centroid coordinates show no strong coupling to any other metabolite in this total data set.

Applying a more stringent threshold of 10^-8 ^(μM s^-1^) μM^-1 ^and an initial seed of 64 cluster centroids results in 45 final clusters encompassing all 4,771 end points. Comparison of cluster centroid sizes and coordinates to those of the 10^-2 ^(μM s^-1^) μM^-1 ^dataset show that trajectories with 1000 μM starch are under-represented at this threshold—each starch concentration represented 8,192 points in the 10^-2 ^(μM s^-1^) μM^-1 ^dataset, since this included all 16,384 data points, whereas there are only 768 end points with 1000 μM starch versus 4,003 end points with 0.1 μM starch at 10^-8 ^(μM s^-1^) μM^-1^. Reaction rates are proportional to substrate concentrations and no starch-based inhibition was present, so the low representation of high-concentration starch trajectories in the end point dataset suggests that these trajectories may have started further from their systems' fixed points in general. On the other hand, samples with lower ATP and orthophosphate concetrations are also selected against; for ATP, there are 3,773 and 998 end points with 1 mM and 0.1 μM fixed concentrations, respectively, and for phosphate 3,334 and 1,437 end points. In this case, rate limitation in the lower-concentration samples may broadly explain the patterns seen. The remaining external metabolites show roughly equal numbers of clusters at each of the two sampled concentrations (see Additional File 1).

Correlation analysis of this smaller, more tightly converged subset of end points shows patterns among stromal starch and maltooligomers similar to those seen for the total dataset. Here, however, arabinogalactan is strongly coupled to the starch-dependent stromal metabolites due to glucose transport and a full correlation of 1000 μM starch glucosyl units with 1.11 mM AG (the lower starch amount of 0.1 does not correlate neatly with any one AG concentration, thus lowering the magnitude of the correlation coefficient). Within this smaller subset of sampled points, the coupling strength between cytosolic and stromal metabolites is larger, and the uniformity of coupling becomes more apparent. This uniformity is likely due in part to the irreversible formulation of the transport reactions, which mimics a cellular situation with high demand for maltose and glucose.

**Figure 14:**
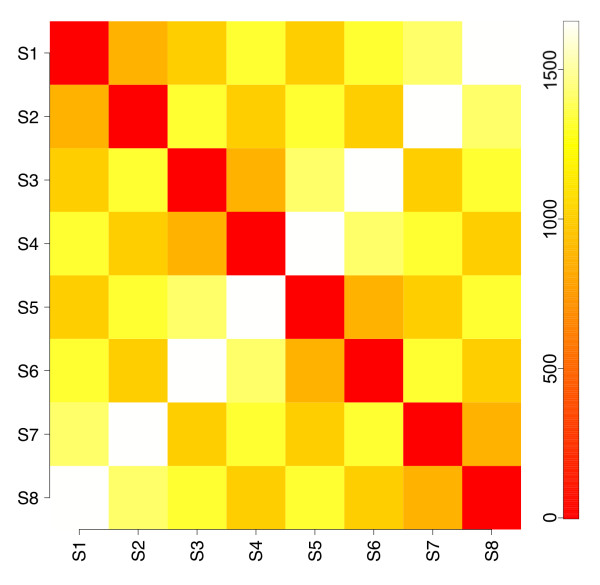
**Euclidean distances between steady states obtained with tight convergence threshold**. Each steady state is conceptualized as a point in the positive orthant of a multidimensional space, with coordinates equal to steady-state concentrations. The numerical threshold for defining a steady state here was 10^-16 ^(μM s^-1^) μM^-1^. Although the distances between different points shown are numerically unique, only 6 are visually distinct due to the compression of small differences by the overall range.

The 213 points representing trajectories with evolution rates less than 10^-16 ^(μM s^-1^) μM^-1 ^were found to cluster into 8 distinct clusters using a sequential clustering algorithm. Mapping the Euclidean distances between them gives rise to an obviously apparent symmetry, seen in Figure 14. Although there are 29 numerically distinct pairwise distances, they visually cluster into only 6 (zero self-distance, 2 edge lengths, and 3 diagonals, represented as raw Euclidean distances, rather than logarithms, due to higher visible resolution). The presence of 8 (2^3^) centroids implies three determining variables; to assess which three metabolites are relevant, the centroid coordinates for all 8 states are examined in Table 3. What is sought is the least number of compounds, or strongly coupled compound groups, that can explain the symmetric array of states. Immediately, species with invariant concentrations may be omitted, as they have no explanatory power; of the remainder, the external cytosolic ADP and glucose 6-phosphate pools clearly define a symmetric quartet of coordinate pairs (0.1, 0.1), (0.1, 1000), (1000, 0.1), and (1000, 1000). Compounds with concentrations that correlate with these two compounds can then be eliminated as determinants of the symmetry, as they are not independent variables (*e.g*., the glucose concentration in row 13 correlates completely with the values of ADP and glucose 6-phosphate). The third symmetry determinant appears to be the metabolically coupled pair of arabinogalactan and the glucosylated form, which have only one independent degree of freedom (*i.e*., their sum is conserved since we neglect biosynthetic or catabolic pathways as mentioned above).

**Table 3:** Steady state concentration vectors for the 8 distinct steady states obtained for tight convergence threshold.

Species/State	S1	S2	S3	S4	S5	S6	S7	S8
1 GlcStarch_CS	1000.0	1000.0	1000.0	1000.0	1000.0	1000.0	1000.0	1000.0

2 Maltose_CS	240.97	240.97	240.97	240.97	240.97	240.97	240.97	240.97

3 Maltotriose_CS	108.52	108.52	108.52	108.52	108.52	108.52	108.52	108.52

4 Maltopentaose_CS	307.79	307.79	307.79	307.79	307.79	307.79	307.79	307.79

5 Glucose_CS	0.52	0.52	0.52	0.52	0.52	0.52	0.52	0.52

6 ATPtot_CY	1000.0	1000.0	1000.0	1000.0	1000.0	1000.0	1000.0	1000.0

7 Pitot_CY	1000.0	1000.0	1000.0	1000.0	1000.0	1000.0	1000.0	1000.0

8 Glc1Ptot_CY	0.1	0.1	0.1	0.1	0.1	0.1	0.1	0.1

9 AG_CY	822.57	1677.08	822.57	1677.08	822.57	1677.08	822.57	1677.08

10 GlcAG_CY	177.53	322.92	177.53	322.92	177.53	322.92	177.53	322.92

11 ADPtot_CY	0.1	0.1	1000.0	1000.0	0.1	0.1	1000.0	1000.0

12 Glc6Ptot_CY	0.1	0.1	0.1	0.1	1000.0	1000.0	1000.0	1000.0

13 Glucose_CY	0.01	0.01	0.02	0.02	0.12	0.12	6.97	6.97

14 Maltose_CY	9.75	10.60	9.75	10.60	9.78	10.62	11.27	11.94


Numerical convergence of integration was 10^-16 ^(μM s^-1^) μM^-1^. Compounds are ordered top to bottom by invariance among states (1-8), explanatory species (9-12), and dependent internal metabolites (13 and 14).

The symmetry observed in this geometric interpretation of steady-state metabolite vectors is identical to that found in the distance matrix of corner positions for a rectangular parallelepiped with two sides equal. This situation arises from two concentrations sampled per compound (0.1 and 1000 μM), and only 4 compounds defining the total range, two of which comprise a dynamically coupled pair (and so provide one effective degree of freedom), giving 2^3 ^= 8 points. ADP and glucose 6-phosphate are external metabolites, and so are fixed at values defining their respective points; the glucosylated arabinogalactan-arabinogalactan pair are internal metabolites. The four states on the corners of the square faces differ in the values of fixed concentrations; the two square faces differ in the third independent determinant's final value. The variability of cytosolic maltose, which slightly perturbs the otherwise perfect symmetry and accounts for the numerical uniqueness of the 29 pairwise distances, illustrates modest sensitivity (9.753 to 11.944 μM) with respect to the much larger ranges of ADP, glucose 6-phosphate, and AG/GlcAG (0.1 to 1000, 0.1 to 1000, and 177.527 to 1677.080 μM). The AG pool size explains the difference in steady-state maltose concentrations between even-and odd-numbered states—the differences between pairs of states (*e.g*., S1/S2 vs. S3/S4 in Table 3) correlate with cytosolic glucose levels. This variation is expected from the reversible cytosolic DPE2 reaction.

Although arabinogalactan and glucosylated arabinogalactan are internal metabolites and therefore free to evolve as the simulation does, their sum is conserved and dictated by their initial concentrations. It was of interest to examine how their sampled initial concentrations correlated with the eight distinct solutions found. In Table 4, one can see that if the initial concentrations of both arabinogalactan and glucosylated arabinogalactan are taken to be 0.1 μM, no trajectories that converge to 10^-16 ^(μM s^-1^) μM^-1 ^are found. However, if one of the pair has an initial concentration of 0.1 μM and the other at 1000 μM, half of the 8 distinct steady states are obtained; the remaining four are found when both initial concentrations are taken as 1000 μM. It is thus the total amount of arabinogalactan (AG + GlcAG) in the system that acts as a third determinant of the symmetry seen in Figure 14—0.2 μM is not represented, 1000.1 μM correlates with the odd-numbered steady states, and 2000 μM with the even-numbered states.

**Table 4:** Trajectory and steady state number versus initial concentrations of arabinogalactan and its glucosylation product.

GlcAG_CY/AG_CY	0.1 μM	1000 μM
**0.1 μM**	357/0	999/70 S1, S3, S5, S7

**1000 μM**	976/77 S1, S3, S5, S7	1057/66 S2, S4, S6, S8

In all these four cases, the initial concentrations of the remaining 6 internal metabolites and the fixed concentrations of the 6 external metabolites are assigned values of 0.1 or 1000.0 μM. The number to the left of a slash is the number of starting trajectories converged to 10^-10 ^(μM s^-1^) μM^-1^, and to the right to 10^-16 ^(μM s^-1^) μM^-1^, from an initial 4096 trajectories. The eight distinct steady states obtained at the latter threshold from sequential clustering analysis are shown as S1,S2,..., S8.

## Discussion

Several observations arise about starch biochemistry and general metabolic simulation from the modeling and simulation of soluble starch degradation kinetics described above. First, reversible β-amylase action on starch should be incorporated to account for lower starch hydrolysis rates upon maltose accumulation, such as that seen in the *A. thaliana mex1 *mutant lacking the maltose transporter (MEX) that accumulates abnormally high levels of maltose and has reduced rates of starch degradation [1,34]. Maltose has also been reported to inhibit some β-amylases at high concentration [35]. Alternatively, the reduction in starch degradation rate in the *mex1 *mutant might arise from multi-oligosaccharides inhibiting an enzyme involved in the attack on the starch granule, possibly by competing with granular starch for a starch-binding domain required for attack on the granule [1,36]. This is supported by experimental observations that the Arabidopsis *dpe1 *mutant, lacking the chloroplastic disproportionating enzyme required for maltotriose metabolism, also exhibits reduced starch degradation rate [1,20]. This mode of inhibition is outside the scope of the model, but is effectively captured by the reversibility of β-amylase kinetics acting on soluble starch.

The model herein is based on the starch degradation pathway postulated by Smith, *et al*. [1], which suggests that starch granules are solubilised to yield soluble branched glucans that are then degraded by debranching and β-amylase enzymes. The mechanism by which the solubilization occurs is not well understood and is likely to involve two dikinases—glucan water dikinase (GWD) [2,3] and phosphoglucan water dikinase (PWD) [4,5]. An alternative pathway might involve the direct attack on the starch granule by β-amylase [1]. Although β-amylase cannot hydrolyze linkages beyond branch points, it could act in tandem with a debranching enzyme to degrade starch granules gradually and directly to maltose and maltotriose. In such a case, the actions of the two dikinases GWD and PWD would determine the extent to which β-amylase can attack chains at the granule surface, since the distribution of the phosphate groups added to amylopectin by these enzymes would reduce the degree of crystalline packing of chains inside the starch granules [37]. Such a pathway would result in formation of solution-phase malto-oligosaccharides directly from insoluble starch granules without the intermediacy of soluble branched and linear glucans. Although earlier studies indicate that β-amylase is incapable of degrading native starch granules [38,39], a chloroplastic β-amylase from potato leaves was recently shown [40] to release malto-oligosaccharides from potato tuber starch granules, which lends credibility to the alternative starch degradation pathway. If β-amylase catalysis of insoluble starch cleavage is possible, the current model can be interpreted as capturing this in an effective way; however, the β-amylase kinetics would need to be re-examined and likely reformulated to accurately capture all the subtlety of this more physically complex process.

A second related conclusion is that flow from *soluble *photosynthetically fixed carbon stores into metabolic pathways of interest in biofuel production is likely primarily limited by the cleavage of the linear polymer to oligomers, and not by subsequent reactions or debranching. This conclusion certainly depends on expression levels and characteristics of enzymes in particular cases, but is supported by our best estimate of biochemically relevant conditions herein, as well as the correlations among simulation end-points representing a reasonably wide range of conditions (100 nM to 1 mM of 8 free initial and 6 fixed concentrations). Experimental investigations have suggested that the solubilisation of starch granules, rather than the hydrolysis of solubilised starch, might constitute the overall limiting step in starch degradation at low temperature [39,41,42]. Although potentially capturing such direct starch cleavage qualitatively as noted above, the model herein focuses on biochemical processes after starch solubilization. The β-amylase rate limitation identified is thus relative to subsequent solution processes only. This conclusion is supported by experimental observations [1,43] that β-amylase activity is strongly correlated to a decrease in starch during fruit ripening in banana plants, and that knockout mutants in *A. thaliana *lacking one of the chloroplastic β-amylases show lowered rates of starch degradation.

A third observation is that transport reactions can serve as a kinetic bottleneck, as seen in the strong negative response coefficients of stromal glucose with respect to the glucose transporter *k_cat _*(Figure 2) and concentration (Figure 8), and strong positive response coefficients versus *K_M _*(Figure 2). Stromal glucose response stands in contrast to that of cytosolic glucose, which is insensitive to any parameters other than the hexokinase equilibrium constant. The latter behavior arises as a consequence of rapid equilibration of cytosolic glucose with the glucose 6-phosphate pool mediated by hexokinase, which mimics rapid glucose flux into downstream carbon sinks. The relative sluggishness of transport can thereby dampen the sensitivity of metabolite concentrations in one compartment from the effects on reactions in another organelle. Such a conclusion is intuitive from topological considerations (two networks are connected by few edges, so requiring perturbations to propagate linearly through at least one reaction step), as well as kinetic ones (to the extent that transport is limiting, fast dynamics on one side of the reaction will not be visible to the other). One can surely devise exceptional cases, and a quantitative elucidation of this statement requires further exploration, but the results here lend support to the validity of a "divide-and-conquer" approach to cellular dynamical studies, with explicit consideration of single membrane-bound compartments or phases coupled by an effective variation of transport flux at the network boundaries, rather than explicitly by large-scale, multicompartment dynamics. It should be noted that the irreversible formulation of transport we have employed naturally limits the degree to which this model can be generalized—situations without strong downstream cytosolic glucose demand would not be well represented, nor special cases in which dynamic glucose or maltose transients in the cytosol occur, since these effects could not be communicated through the transporters to the stromal metabolite pools.

A fourth conclusion may be summarized as a critique of what appears to be an implicit assumption that a single steady state resembling a handful of observations is necessarily the most important. To the degree that enzyme kinetics measured *in vitro *reflects turnover response to metabolite concentrations *in vivo*, kinetic models similar to that presented here are large dynamical systems linear in flux, but nonlinear in concentration. Such dynamical systems potentially possess great complexity, not only with respect to bifurcations as parameters vary, but also with respect to the phase space of concentrations under a single assumed set of parameters. A system infinitely "robust" with respect to temporally varying metabolite concentrations would indeed evolve toward a single unique steady state starting from anywhere in the relevant phase space dictated by catalyst concentrations and kinetic properties. The system explored herein behaves robustly with respect to the initial concentrations of internal metabolites, and with respect to perturbation of kinetic parameters and enzyme concentrations. Changing the latter for β-amylase and MEX by up to 10-fold resulted in quantitatively similar steady-state response coefficients. From a biological control perspective, this robustness is desired—variation of enzyme levels arising from genetic regulation or post-translation modification should not give rise to catastrophic divergence of the cellular state. This robustness is also favorable for evolutionary or *in vitro *metabolic engineering, in that changing the nature of the phase space by mutagenic kinetic parameter variation will not lead to lethal cellular phenotypes. Nevertheless, given the complexity of biochemical regulation and chemical dynamics, we expect some surprises as biochemical models grow in fidelity, high-performance simulation and advanced analytical tools become available, and cross-disciplinary fertilization occurs between biochemists and mathematicians with interests in system dynamics.

Simulation of broadly sampled model concentrations and subsequent trajectory end-point analysis showed correlation patterns consistent with the topology of the model metabolic network. This approach was found to be useful in identifying certain relationships, such as tight coupling of stromal metabolism, relative convergence values between classes of simulations (high and low starch, low and high ATP and orthophosphate), and sensitivity of internal metabolite concentrations (steady-state maltose concentration dependence on arabinogalactan and glucose levels in the cytosol). Although computational studies of large-scale kinetic systems of the complexity found in cells is still in its infancy, the dual contemporary interests in parallel and data-intensive computing is opening the door to discovering unforeseen behaviors and patterns in biochemical networks. The work here has only touched on the full content of even this small metabolic model—simply exploring the appropriate cellular context by varying bath metabolite pool sizes, and potential multiplicity of fixed points by varying initial internal metabolite concentrations, generates a substantial quantity of data requiring significant analysis effort. Nevertheless, bringing metabolic engineering on par with traditional engineering disciplines will require thorough quantitative understanding of both system dynamics, and the effects of parametric variability beyond values that nature provides. This transformation will be facilitated by further development and adaptation of analytical and visualization methods for biochemical systems analysis.

## Conclusions

Construction and characterization of a kinetically detailed model of starch metabolism shows that β-amylase activity is the limiting factor in saccharide production under conditions of high glucose demand, using best estimates for kinetic parameters and enzyme levels. Sensitivity analyses and sampling of internal and external metabolite concentrations and clustering analysis of fixed-time simulation endpoints showed that soluble starch levels are the main determinant of debranched starch and maltooligomer levels in the chloroplast stroma, but that transport reactions partially decouple the cytosolic chemical subsystem directing carbon flow to downstream sinks. The most tightly converged end points illustrate a role for the metabolically coupled arabinogalactan/glucosylated arabinogalactan pair and cytosolic glucose levels in determining steady state maltose levels. No evidence for multistationarity was found. The model and explorations described highlight areas in starch metabolism for deeper study and experimental testing, as well as potential opportunities for methodological advancement.

## Methods

### Model Formulation: Nomenclature of Metabolites, Enzymes, Transporters, and Parameters

Compound abbreviations are defined in Tables 1 and 2. The intracellular compartment in which a compound or enzyme resides is denoted by a two-letter suffix preceded by the underscore sign, with "_CY" denoting cytosolic and "_CS" a chloroplast stromal species. Where ionization is possible, pools of ionizable species containing all the biologically occurring ionized and un-ionized forms are appended with a "tot" subscript. For example, the pool of phosphate in the cytosol is represented as Pitot_CY. For polymeric starch, when a single residue of the polymer—the starch glucosyl unit—constitutes a separate model entity, the residue identity is represented as GlcStarch_CS. The aggregate pool of linear linkage groups released from solubilized starch by the action of the debranching enzyme is assigned the abbreviation Starch_db__CS. The enzymes are usually represented using their Enzyme Commission (EC) numbers, such that the enzyme names consist of the prefix "ec_" followed by the EC number with the dots substituted with underscores. Thus, the chloroplastic form of the disproportionating enzyme 1 DPE1 with EC number 2.4.1.25 is therefore represented as ec_2_4_1_25_CS.

Kinetic parameters are generally referred to with appropriate formatting in the text, *e.g*., β-amylase  for the turnover number of β-amylase acting on starch as a substrate. For figure labels, an alternative nomenclature was used for simplicity in formatting. Thus, β-amylase  is referred to as "betaAmylase_Gn_kcat". Differing parameters for alternative substrates are denoted by superscripted parentheticals, so the k_cat _parameters for starch (Gn) and maltopentaose (G5) degradation by β-amylase are denoted by  and , respectively. The names of the enzyme kinetic parameters and equilibrium constants for the cytosolic disproportionation catalyzed by DPE2 all start with the locus tag for this enzyme in *Arabidopsis thaliana*, AT2G40840, rather than the KEGG [44] reaction identifier.

To treat transport reactions between compartments of different volumes consistently, all reaction rate equations carry the appropriate volumetric factors in the model (see Additional File 2). Thus, each rate-of-change is calculated as a mass rate-of-change rather than a concentration rate-of-change, consistent with the SBML standard [45]. However, we have excluded these factors in the Tables to be more consistent with standard biochemical nomenclature.

### Model Formulation: Biochemical Processes

#### Degradation of starch to maltose and maltotriose

Degradation of solubilized starch to maltose and maltotriose in the chloroplast stroma is modeled by a modified version of the kinetic expression proposed by Shiraishi, *et al*., to describe maltose production from soluble starch by the concerted use of β-amylase and debranching enzymes [29]. The total mass concentration of starch is divided into two parts as seen in Figure 10 of Ref. [29], namely that hydrolyzable solely by β-amylase *S_β0 _*and that requiring both β-amylase and a debranching enzyme for hydrolysis, *S_df_*. Using the representation scheme in this figure, the total mass concentration hydrolyzed solely by β-amylase is

and the total mass concentration hydrolyzed by the combined action of β-amylase and debranching enzyme is given by

*f_β _*= 0.582 is defined as the mass fraction of starch that can be degraded by the action of β-amylase alone.

The rate equations of maltose and maltotriose formation resulting from starch degradation are given in Table 5. The mass concentration of linear linkage groups released from starch produced by the action of a debranching enzyme is defined as *S_db_*, the time evolution of which was modeled after [29] and is also detailed in Table 5. The initial concentration of such groups is set to 0 at *t *= *0*. Fractions *f_M _*= 0.87 and *f_G3 _*= 0.13 represent starch ultimately convertible to maltose and maltotriose, respectively.

**Table 5:** Kinetic equations and parametric assignments for β-amylase.

Maltose formation from soluble starch	
Maltotriose formation from soluble starch	

Release of linear linkage groups from starch^1^	

Maltopentaose degradation to Maltose and Maltotriose	

The kinetic model includes formation of maltose and maltotriose from starch and maltopentaose. β-Amylase turnover numbers  and , mass concentration *E_β_*, equilibrium constant between maltose and maltotetraose *K_eq_*, Michaelis constants  and , inhibition constant associated with maltotetraose formation , mass concentration of debranching enzyme *E_d_*, and rate constant for debranching *k_d _*are taken from reference [29]. *M *denotes the mass concentration of maltose, and terms containing *M^2 ^*are related to β-amylase-catalyzed maltose condensation to yield maltotetraose.

^1 ^*k_d _*is bi-valued, with  for *S_db_*/*S_df _*< 0.3, and  for *S_db_*/*S_df _*≥ 0.3.

#### Disproportionation reactions

The disproportionation of two maltotriose molecules to glucose and maltopentaose is catalyzed by disproportionation enzyme 1 (DPE1). The relevant equations and parameters for this biochemical reaction adapted to a single substrate pool are shown in Table 6. The cytosolic transglucosidase (DPE2) reaction splits maltose into two glucosyl units, one of which ends up as free glucose and the other transferred to a heteroglycan (arabinoglycan) acceptor to yield glucosylated arabinogalactan. Both reactions were modeled with Ping-Pong BiBi kinetics [30], because many enzymatic disproportionation reactions have been reported in the literature to proceed by this mechanism [46-48]. Kinetic equations and parameters are given in Tables 6 and 7.

**Table 6:** Kinetic equations for maltotriose disproportionation to glucose and maltopentaose by DPE1.


Haldane relation 1	[30]

Haldane relation 2	[30]

Haldane relation 3	[30]

K_eq_	1.0 [53]

K_MA_	3.3 mM [54]

V_fmax_	; *[DPE1] *= 2 μM

K_MP_^1^	11.7 mM [55]

K_MQ_^2^	0.21 mM [55]

K_iQ_^3^	0.1 mM [55]

K_iP_^4^	5.57 mM

K_iA_^5^	746 μM

Concentrations *A*= maltotriose, *P *= glucose, and *Q *= maltopentaose.

^1 ^The *K_M _*(glucose) value was taken from that for the disproportionation reaction between 4-nitrophenyl-α-D-maltoheptaoside-4-6-O-ethylidene (EPS) as donor and glucose as acceptor catalyzed by the cyclodextrin glycosyltransferase (CGTase) enzyme in *Thermoanaerobacterium thermosulfurigenes*.

^2^Substituted by the *T. thermosulfurigenes K_M _*(EPS) value for the disproportionation reaction between EPS as donor and maltose as acceptor catalyzed by the *T. thermosulfurigenes *CGTase.

^3^Taken as 0.1 × the competitive *K_i _*(γ-cyclodextrin) value for the disproportionation between EPS as donor and maltose as acceptor catalyzed by *T. thermosulfurigenes *CGTase.

^4^From Haldane relations 1 and 2.

^5^From Haldane relation 3.

**Table 7:** Kinetic equations for maltose disproportionation to glucose and glucosylated arabinogalactan by DPE2.


Haldane relation 1	[30]

Haldane relation 2	[30]

*K_eq_*	1.0 [53]

*K_MA_^4^*	4.6 mM [55]

*K_MB_^1^*	1.1 mM [55]

*V_fmax_^3^*	; *[DPE2] *= 2 μM

*K_MP_^3^*	11.7 mM [55]

*K_MQ_*^1^	1.1 mM [55]

*K_iB_^2^*	1.0 mM [55]

*K_iQ_*^2^	1.0 mM [55]

*K_iP_*^3^	5.57 mM

*K_iA_*^5^	2.19 mM

Concentrations *A*= maltose, *B *= arabinogalactan, *P *= glucose, and *Q *= glucosylated arabinogalactan.

^1 ^The *K_M _*(arabinogalactan) and *K_M _*(glucosylated arabinogalactan) values were taken from the disproportionation reaction between 4-nitrophenyl-α-D-maltoheptaoside-4-6-O-ethylidene (EPS) as donor and maltotetraose as acceptor catalyzed by the cyclodextrin glycosyltransferase (CGTase) enzyme in *Thermoanaerobacterium thermosulfurigenes*.

^2^Substituted by the *T. thermosulfurigenes K_i, competitive _*(γ-cyclodextrin) value for the disproportionation reaction between EPS as donor and maltose as acceptor catalyzed by the *T. thermosulfurigenes *CGTase.

^3^Taken from the DPE1 model.

^4 ^Taken from *T. thermosulfurigenes K_M _*(maltose) value for the disproportionation reaction between EPS as donor and maltose as acceptor catalyzed by the *T. thermosulfurigenes *CGTase.

^5^From Haldane relations 1 and 2 and values for associated kinetic constants given.

#### Degradation of maltopentaose by β-amylase

The degradation of maltopentaose formed by the chloroplastic disproportionation reaction is modeled as

where *G_5 _*denotes the maltopentaose mass concentration, *E_β _*represents β-amylase mass concentration, and  and  represent the turnover number of the β-amylase enzyme for maltopentaose degradation and the Michaelis constant for maltopentaose, respectively [29].

#### Transport reactions

The present model includes two catalyzed transport processes from chloroplast stroma to cytosol. Both are modeled as irreversible Michaelis-Menten processes,

with *S *being either chloroplastic maltose or glucose, and *V_max _*factorable as *k_cat _*× [*transporter*]. The value of  is calculated as 11.9 μM s^-1 ^using the maximal maltose consumption rate for a given residual maltose concentration and the mass concentration of cells in anaerobic sugar-limited yeast (*Saccharomyces cerevisiae *CBS 8066) chemostat cultures [49]. Based on a value for  of 519 μmole (mg of chlorophyll hr)^-1 ^[21] and the equivalence between 1 mg of chlorophyll to 30 μL of chloroplast stroma from [50], we obtain  = 4806 μM s^-1^_. _Parameters are given in Table 8.

**Table 8:** Kinetic factors for maltose and glucose transport between chloroplast stroma and cytosol.

Parameter	Maltose	Glucose
*k_cat_*^1^	5.96 s^-1^	240.28 s^-1^

[*transporter*]^1^	2 μM	20 μM

*K_M_*	4 mM[49]	19.3 mM[21]

^1^Rate constant and transporter concentrations are derived from *V_max _*values as described in the text. Transporters are given plausible concentration values.

Release of Glc-1-P from glucosylated heteroglycan and hexokinase The cytosolic glucan phosphorylase (glycosylated heteroglycan → glucose-1-phosphate) was modeled as a rapid-equilibrium random Bi-Bi mechanism [30,51]. The velocity expression for this reaction is given in Table 9. Cytosolic hexokinase forming glucose-6-phosphate from glucose and ATP is modeled similarly using the same velocity equation and enzyme kinetic and binding parameters as in the model of human erythrocyte hexokinase developed by Mulquiney and Kuchel [51].

**Table 9:** Kinetic equation and factors for cytosolic glucan phosphorylase.


Haldane relation	

Equilibrium constant from substrate and product Gibbs free energies^1^	

Gibbs reference free energy corrected for ionic strength [56]	

*V_fmax_*	; [*CGP*] = 2 μM

*K_MA_^2^*	2.1 mM[57]

*K_iA_^2^*	3.8 mM[57]

*K_MB_^2^*	5.9 mM[57]

*K_MP_^2^*	2.0 mM[57]

*K_iP_^2^*	3.1 mM[57]

*K_MQ_*	3.8 mM[57]

Concentrations *A *= glucosylated arabinogalactan, *B *= phosphate, *P *= glucose-1-phosphate, and *Q *= arabinogalactan.

^1 ^is the standard Gibbs free energy of formation of species *i *with charge *z_i _*at 298.15 °K and at a cytosolic ionic strength (I) of 0.15 M. The (*I = 0*) values for *A *(glucosylated arabinogalactan) and *Q *(arabinogalactan) are assumed to be equal to the corresponding values for Glycogen(n) and Glycogen(n-1) respectively tabulated in Ref. [58]. The (*I = 0*) values for *B *(phosphate) and *P *(glucose-1-phosphate) are tabulated in Ref. [56].

^2 ^*K_mA_*, *K_iA_*, and *K_MB _*values are taken from the polysaccharide degradation reaction catalyzed by potato phosphorylase, with *A *= amylopectin and *B *= phosphate. The *K_MP_*, *K_iP_*, and *K_MQ _*values are taken from the polysaccharide synthesis reaction catalyzed by potato phosphorylase, with *P *= glucose 1-phosphate and *Q *= amylopectin.

The rate-of-change expressions for individual metabolites in terms of reaction fluxes are contained in Additional File 3.

### Simulation Framework and Methodology: Model instantiation and simulation

The starch degradation model was expressed in Systems Biology Markup Language (SBML) [45] and input into our metabolic simulation software framework, the High-Performance Systems Biology Toolkit (HiPer SBTK) [52]. The time evolution of metabolite concentrations and fluxes was simulated, and the possible space of rate and binding parameters sampled. Biologically feasible concentrations of internal metabolites and fixed concentrations of external metabolites, enzymes, transporters and inhibitors were imposed, then the ODE system integrated. Sensitivities of steady-state internal metabolite concentrations to enzyme concentrations and kinetic parameter values were then computed. Additionally, the structure of the dynamical space was explored by sampling initial concentrations of internal metabolites given a fixed set of enzyme and inhibitor concentrations and kinetic parameter values, and evaluating the distance between steady states so achieved.

A reference system was defined by starting from a best estimate of biologically relevant metabolite, enzyme, transporter and inhibitor concentrations (Tables 1 and 2). This model was integrated for 10^7 ^virtual seconds to a convergence of 1.6 × 10^-13 ^(μM/s) μM^-1^. Response coefficients [31] of the internal metabolite steady-state concentrations *S_i _*with respect to the kinetic and binding parameters and fixed enzyme and transporter protein concentrations *p_j _*were calculated. In addition, "parameter-perturbed" and "enzyme-perturbed" systems were modeled by decreasing  and  10-fold relative to the reference model in the former case, and increasing β-amylase concentration two-fold and the maltose exporter concentration ten-fold in the latter. Integrations of the parameter-and enzyme-perturbed models converged to 1.5 × 10^-16 ^and 5.2 × 10^-12 ^(μM/s) μM^-1^, respectively.

Additional File 3 contains the integration timecourse for the reference model, close-up of debranched stromal starch evolution, and a graphical summary of the response coefficients and norms for the 8 states detailed in Table 3.

### Analysis of States from Varying External or Initial Internal Metabolite Concentrations

To evaluate both the existence of multiple stationary states within individual dynamic systems, and the effects of external metabolite concentrations on the fixed point(s) of the modeled biochemical network, 6 external and 8 initial internal metabolite concentrations were sampled at two values, and in each of the 2^14 ^cases integrated as described above. Each ODE integration was run for 10^7 ^virtual seconds, and convergence of each trajectory quantified as

where [*S_i_*] is the concentration of metabolite *i*, and C carries units of (μM/s) μM^-1^. Final metabolite vectors at convergence cutoffs of 10^-2 ^and 10^-8 ^were clustered by the k-means algorithm (as implemented in the SciPy package). Each row of the data matrix comprised a final point in the logarithm of metabolite concentration space. The variance of each column was normalized to 1.0, then the data clustered with a seed cluster estimate of 64 (2^6^, for two sampled fixed concentrations of six external metabolites). Cluster centroid distances were checked to be greater than the sum of their respective cluster standard deviations. Establishing unit variance for the most tightly converged metabolite vectors was problematic, and so data in this case were clustered sequentially in the direct space of concentrations, using a distance threshold of 10^-8 ^μM. For this latter case, a cluster could be defined by the position of the first point added, rather than the centroid, due to the relatively tight bunching observed.

## Competing interests

The authors declare that they have no competing interests.

## Authors' contributions

AN developed and encoded the starch degradation model, ensured model self-consistency, simulated the model behavior, analyzed results from serial and parallel simulations, and drafted the manuscript. ML implemented the parallel parameter sampling routines and carried out parallelized simulations. PG wrote the sequential clustering routine, supervised the software design and implemented the core simulation components. CHC developed the modeling strategy, wrote and ran the k-means clustering routine and analyzed the results, co-drafted and edited the manuscript, and supervised the research. All authors have read and approved the final manuscript.

## Authors' information

AN is a research associate in the Computational Science Center at the National Renewable Energy Laboratory (NREL) with research interests including mathematical biology and theoretical immunology. His current explorations include metabolome representation and expression for potential biofuel-producing organisms, enzymatic hydrolysis of biomass particles containing multiple polysaccharide types, flux balance analysis, and modeling regulatory networks using data-mining, bioinformatics analysis and correlation of multiple types of high-throughput "omics" data.

ML is a research associate in the Computational Science Center at NREL. His research interests are numerical search, high performance computing (HPC) and uncertainty quantification. In addition to supporting systems biology, Monte is also working on exploring more efficient building designs using parallel computing.

PG is a senior scientist in the Computational Science Center at NREL. His research interests center on the application of mathematics and HPC to renewable energy challenges, particularly optimizations involving expensive simulations. In addition to developing kinetic simulation software for HPC, he studies multi-scale device simulation and optimization and high-throughput materials screening, inverse material design by electronic structure data mining and optimization, and computational battery modeling.

CHC is a senior scientist in the Computational Science Center at NREL. His research focuses on systems biochemistry and computational chemistry from molecular to cellular scales. His current projects touch on dynamical properties of chemical and biochemical systems, electron and energy transfer, and homogeneous transition metal catalysis.

## Supplementary Material

Additional file 1 **Clustering analysis**. A single Excel 2008 file containing statistics about k-means clusters for 10^-2 ^convergence cutoff with 63 clusters, and 10^-8 ^convergence cutoff with 45 clusters. Correlation coefficients are also contained as separate sheets within the file, color-coded by magnitude of coefficient: gray (1.0), orange (0.5-0.99), green (0.2-0.49), and white (< 0.2). Click here for file

Additional file 2 **SBML Model File**. A single Systems Biology Markup Language file representing the reference model in this study. This model is automatically transformed to C++ by the translation utility of the High-Performance Systems Biology Toolkit and compiled into high-performance executable programs for sampling and optimization tasks, as well as simple forward integration. This model may also be found in the BioModels database under accession number MODEL1106030000. Click here for file

Additional file 3 **Summary graphics and enzyme kinetic models**. Integration timecourse for the reference model, close-up of debranched stromal starch evolution, a graphical summary of the response coefficients and norms for the 8 states detailed in Table 8 and rate-of-change equations in terms of reaction fluxes. Click here for file
