# Supplementary material for: Kinetic modeling and exploratory numerical simulation of chloroplastic starch degradation
Source: BMC Syst Biol. 2011 Jun 18;5:94. doi: 10.1186/1752-0509-5-94 (PMC3148208; doi:10.1186/1752-0509-5-94)
Supplement: Additional file 3 — Summary graphics and enzyme kinetic models. Integration timecourse for the reference model, close-up of debranched stromal starch evolution, a graphical summary of the response coefficients and norms for the 8 states detailed in Table 8 and rate-of-change equations in terms of reaction fluxes. [file 1752-0509-5-94-S3.DOCX]

## Figure S1 - Temporal profiles of active metabolite concentrations during simulation to steady state.

Solution of the governing ordinary differential equation system, characterized by parameters in Table 1, is treated as an initial value problem, with integration starting from biologically feasible initial concentrations given in Table 2. The system of ordinary differential equations governing the enzyme-catalyzed and transporter-mediated kinetics is integrated over time until the concentrations of the metabolites reach steady state, i.e., do not effectively change with time. This figure shows the temporal evolution of several metabolite concentrations from non-zero initial concentrations. As mentioned in the text the steady state represented in this figure is referred to as the reference steady state or reference state.


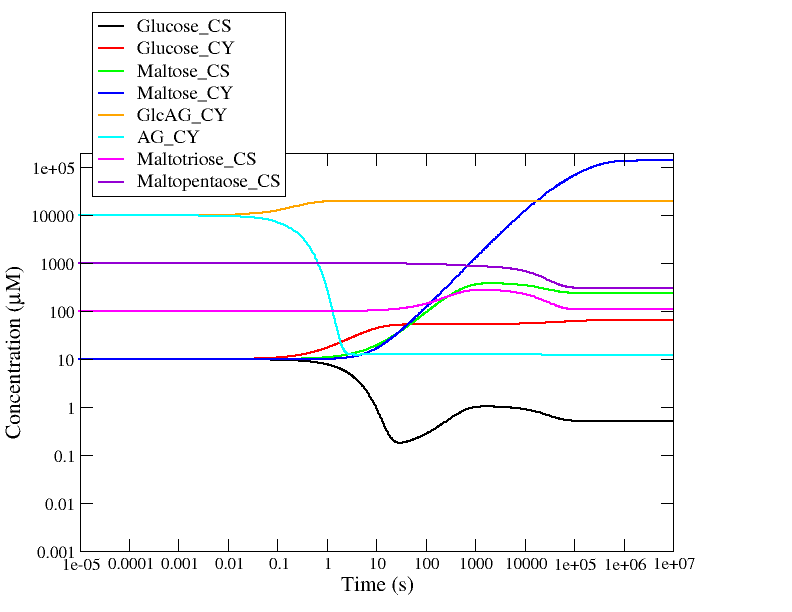


## Figure S2 - Temporal profile of linear starch linkage groups during simulation to steady state.

Linear starch linkage groups are released by the action of the debranching enzyme. The initial concentration of these linear linkage groups is zero and the maximum concentration is 0.418 of total starch corresponding to the known accessible fraction of starch. The underlying formation kinetics of these linkage groups is therefore modeled in such a way (see footnote of Table 3) that the rate of formation drops to zero as its concentration reaches this limiting fraction. The total fixed starch concentration is 0.162 gm⋅L^-1^ by virtue of the starch degree of polymerization (n=1667) and molecular weight (0.27 gm⋅µmole^-1^). The final value of the starch linear linkage group concentration should be 0.162 gm⋅ L^-1^ × 0.418 = 0.0677 gm⋅ L^-1^.


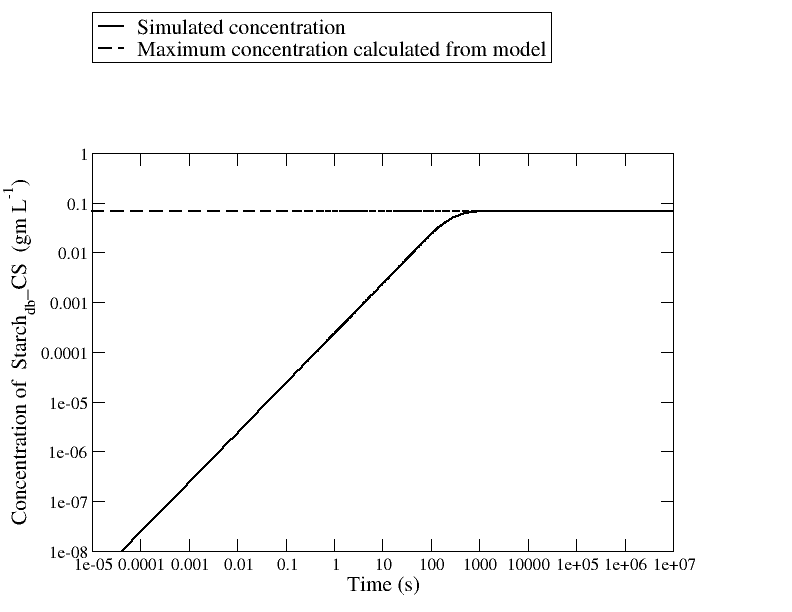


## Figure S3 – Illustration of tetragonal symmetry in the steady state distance matrix and its relevance to points in metabolite concentration space @ C = 10^-16^ (μM⋅s^-1^)⋅μM^-1^.

The corners of the parallelepiped represent the eight points in metabolite concentration space analyzed in Table 8 of the manuscript, and are numbered accordingly. The points are determined by three factors: the fixed concentrations of ADP and glucose-6-phosphate, which are external metabolites in the model presented, and the sum of arabinogalactan and glucosylated arabinogalactan concentrations, which is also a conserved quantity in the simulations. Although the range of the sum AG + GlcAG (2000 – 1000.1 = 999.9) present in the eight centroids discussed is the same as that for ADP and Glc6P (1000 – 0.1 = 999.9), the actual positions of the points in the 14-dimensional space of metabolites is dictated by the particular values of AG and GlcAG, which fall into two pairs (822.57 + 177.53 and 1677.08 + 322.92, see Table 8); thus, the symmetry is only tetragonal. We have distinguished this subtlety from the simple sum as {AG,GlcAG}^+^ in the figure. The five lines of equivalent distances in the upper part of the figure correlate with the five colors seen in the upper triangular region of the distance matrix of Figure 14.


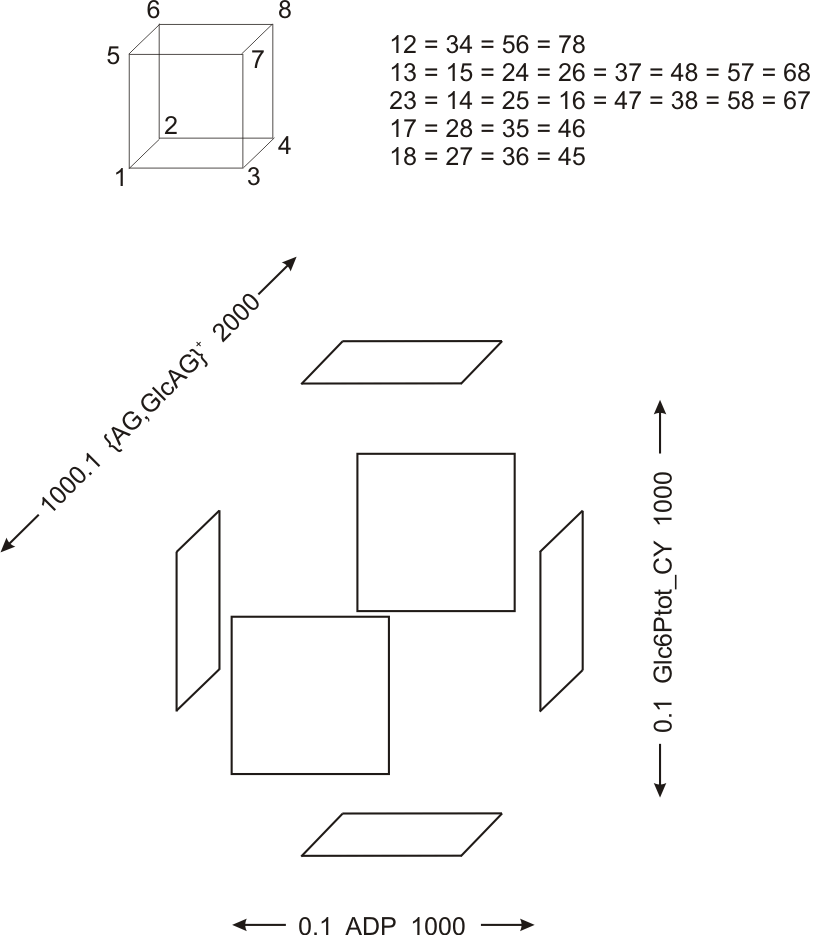


**Rate equations of the variable metabolite species in the model in terms of reaction fluxes**

$$\frac{d[Maltose\_CY]}{dt}=\nu_{MEX}-\nu_{DPE2}$$

$$\frac{d[Maltose\_CS]}{dt}={fc}_{G2}v_{M,\beta-amylase}+ {fc}_{G2}v_{G5,\beta-amylase}- \nu_{MEX}$$

$$\frac{d[Maltotriose\_CS]}{dt}= {fc}_{G3}v_{G3,\beta-amylase}+ {fc}_{G3}v_{G5,\beta-amylase}-2\nu_{DPE1}$$

$$\frac{d[Maltopentaose\_CS]}{dt}=-{fc}_{G5}v_{G5,\beta-amylase}+\nu_{DPE1}$$

$$\frac{d[Glucose\_CS]}{dt}=\nu_{DPE1}- \nu_{pGlcT}$$

$$\frac{d[Glucose\_CY]}{dt}= \nu_{pGlcT}+ \nu_{DPE2}-\nu_{HexK}$$

$$\frac{d[AG\_CY]}{dt}=-\nu_{DPE2}+\nu_{CGP}$$

$$\frac{d[GlcAG\_CY]}{dt}=\nu_{DPE2}-\nu_{CGP}$$

The rate expression for the formation of debranched starch, which is the same as the rate-of-release of linear linkage groups from starch, has been provided in Table 2. The concentration rates-of-change of each metabolite in the above equations have units of µM⋅s^-1^. The factors ${fc}_{G2}$, ${fc}_{G3}$ and ${fc}_{G5}$ correspond respectively to conversion factors (from gm⋅(L^-1^s^-1^) units to µM⋅s^-1^ units) for Maltose_CS, Maltotriose_CS and Maltopentaose_CS flux terms containing $v_{M,\beta-amylase}$, $v_{G3,\beta-amylase}$and $v_{G5,\beta-amylase}$ since the latter three reaction fluxes have gm⋅(L^-1^s^-1^) units in the model. The values of ${fc}_{G2}$, ${fc}_{G3}$ and ${fc}_{G5}$ are respectively 2924, 1984 and 1208. Full expressions for individual flux terms other than $\nu_{MEX}$, $\nu_{pGlcT}$ and $\nu_{HexK}$ have been provided in the tables. $\nu_{MEX}$ (MEX-catalyzed maltose transport) and $\nu_{pGlcT}$ (pGlcT-catalyzed glucose transport) have been specified in the model formulation part of the Methods section, and the values of the related parameters in Table 6; $\nu_{HexK}$ (hexokinase) is equated to the right-hand side of Eq. A1 in Ref. 50.
